# A high‐calcium diet failed to rescue an osteopenia phenotype in claudin‐18 knockout mice

**DOI:** 10.1002/phy2.200

**Published:** 2014-01-13

**Authors:** Fatima Z. Alshbool, Catrina Alarcon, Jon Wergedal, Subburaman Mohan

**Affiliations:** 1 Musculoskeletal Disease Center, Jerry L Pettis VA Medical Center, Loma Linda, 92357, California; 2 Department of Pharmacology, Loma Linda University, Loma Linda, 92354, California; 3 Department of Medicine, Loma Linda University, Loma Linda, 92354, California; 4 Department of Biochemistry, Loma Linda University, Loma Linda, 92354, California; 5 Department of Physiology, Loma Linda University, Loma Linda, 92354, California

**Keywords:** BMD, bone resorption, calcium, claudin‐18, gastric pH

## Abstract

We have recently demonstrated that mice with disruption of claudin‐18 (Cldn‐18) gene exhibited osteopenia due to increased bone resorption (BR). In this study, we found that gastric pH was significantly higher in Cldn‐18 knockout (KO) mice compared to heterozygous control mice at 10 weeks of age. To test the possibility that the increased BR in the Cldn‐18 KO mice fed a normal‐Ca diet is a consequence of decreased Ca absorption caused by increased stomach pH, we subjected KO and control mice to a normal‐Ca and high‐Ca diet at birth. Serum Ca levels were significantly lower in Cldn‐18 KO mice compared to control mice at a normal‐Ca diet but not at high‐Ca diet. Dual energy X‐ray absorptiometry revealed that a high‐Ca diet significantly increased lumbar bone mineral density (BMD), but had no effect on femur/tibia BMD in both Cldn‐18 KO and control mice compared to a normal‐Ca diet. While a high‐Ca diet did not affect volumetric BMD, trabecular, and cortical parameters of the lumbar vertebra (LV) as measured by *μ*CT, the size of the LV did increase, in both genotypes due to reduced BR. Comparison of the skeletal phenotype of high‐Ca Cldn‐18 KO and control mice revealed that an osteopenia phenotype seen at a normal‐Ca diet was still maintained at different skeletal sites in the KO mice till 10 weeks of age. In conclusion, our findings suggest that increased BR is likely caused by direct effects of a lack of Cldn‐18 on osteoclasts rather than gastric pH changes.

## Abstract (precis)

In this study, we examined if calcium deficiency caused by low‐gastric acidity was the cause of osteopenia phenotype observed in claudin‐18 (Cldn‐18) knockout (KO) mice. Our findings that high‐calcium diet did not rescue the osteopenia phenotype in Cldn‐18 KO mice suggest that increased bone resorption is likely caused by direct effects of a lack of Cldn‐18 on osteoclasts rather than gastric pH changes.

## Introduction

Osteoporosis is a major clinical problem in which loss of bone strength leads to skeletal fractures (Dempster 2011). Osteoporosis‐mediated bone fragility can result from failure to produce optimal bone mass and/or the imbalance between bone formation and bone resorption (BR) during bone remodeling (Raisz 2005). It is well‐known that BR is dependent on the number and activity of osteoclasts, which is in turn is regulated by a number of local and systemic factors including macrophage colony‐stimulating factor (MCSF) and receptor activator of nuclear factor kappa B ligand (RANKL). While the MCSF and RANKL are sufficient for inducing osteoclast activity and function, the role of other regulatory molecules and their signaling pathways has not been determined (Boyle et al. 2003; Kearns et al. 2008).

Tight junctions (TJs) play important roles in different biological systems (Chiba et al. 2008). Claudins (Cldns) comprise a major group of TJ proteins and consist of 27 members in mice and humans (Matter and Balda 2007; Krause et al. 2008; Mineta et al. 2011). Cldns serve as a multifunctional complex: they regulate paracellular transport of ions, solutes, and water; and serve as a fence that divides apical and basolateral domains of plasma membranes. Moreover, Cldns act noncanonically by regulating a variety of signaling molecules that control cell differentiation, proliferation, and polarity (Matter and Balda 2007; Chiba et al. 2008; Krause et al. 2008; Steed et al. 2010). Given that Cldns have major functions in different biological systems, the role of individual Cldns has been investigated by employing loss of function models in vivo, and by transgenic studies in cells in vitro (Furuse 2009). However, the role of Cldns in bone homeostasis is still poorly defined. Nonetheless, we have recently demonstrated that one of the Cldns, namely, Cldn‐18 is expressed in bone and that targeted disruption of Cldn‐18 in mice resulted in markedly decreased total body bone mineral density (BMD), trabecular volume, and cortical thickness (Linares et al. 2012).

In our previous studies, we provided the first evidence that Cldn‐18 is expressed in osteoclasts and that the underlying cause of decreased bone mass observed in Cldn‐18 knockout (KO) mice was increased BR, but not impaired bone formation (Linares et al. 2012). We investigated the mechanism by which loss of Cldn‐18 increased BR and found that osteoclast differentiation was increased by regulating RANKL signaling (Linares et al. 2012). The noncanonical effect of the loss of Cldn‐18 on RANKL actions was shown to be mediated by disruption of the interaction with a scaffold protein called zonula occludens (ZO)‐2 that resulted in increased nuclear translocation of ZO2 (Linares et al. 2012). In turn, this translocation increased the expression of important transcription factors involved in RANKL‐induced osteoclast differentiation (Linares et al. 2012). As Cldn‐18 was globally disrupted and Cldn‐18 is expressed in other tissues, the purpose of this study was to investigate the possibility that the osteopenia phenotype of Cldn‐18 KO mice is mediated in part by disruption of Cldn‐18 in other tissues, that is, the stomach (Krause et al. 2008; Tureci et al. 2011).

It has been found that Cldn‐18 is the predominant form of Cldns in the stomach, and has been shown to play a major role in the physiology and pathology of the stomach epithelial barrier (Hayashi et al. 2012; Tamura et al. 2012). The expression level of stomach Cldn‐18 is significantly downregulated in atrophic gastritis and gastric cancer in humans (Sanada et al. 2006). Moreover, targeted disruption of Cldn‐18 in mice resulted in abnormalities in gastric mucosa and atrophic gastritis via decreasing paracellular barrier against H^+^ in stomach epithelium (Hayashi et al. 2012). Interestingly, several clinical studies have shown that gastric acidity is important for calcium absorption in the small intestine (Wright et al. 2008). In this regard, we observed that serum parathyroid hormone (PTH) levels were elevated in Cldn‐18 KO mice fed a normal‐calcium diet compared to control mice, thus suggesting that Cldn‐18 KO mice may be calcium deficient. Based on these findings, we undertook studies to characterize the impact of a lack of Cldn‐18 on the gastric pH and serum calcium levels, and whether a dietary manipulation of calcium homeostasis can rescue the osteopenia phenotype in the Cldn‐18 KO mice. Collectively, our findings revealed that a high‐calcium diet failed to rescue an osteopenia phenotype in Cldn‐18 KO mice.

## Materials and Methods

### Animals

The generation of Cldn‐18‐deficient mice by homologous recombination and genotyping was previously described (Linares et al. 2012). As we did not observe any apparent differences in the skeletal phenotype between heterozygous and wild‐type (WT) animals, we opted to use the heterozygous littermates as control mice. Homozygous Cldn‐18 KO and heterozygous littermate controls were generated by breeding heterozygous with homozygous Cldn‐18 KO. Mice were housed at Jerry L. Pettis Memorial VA Medical Center Medical Unit (Loma Linda, CA) under standard approved laboratory conditions. All animal experiments were performed in compliance with and approved by the Institutional Animal Care and Use Committee.

### Experimental design

Cldn‐18 KO and heterozygous control mice were subjected to either normal‐calcium diet or high‐calcium diet by feeding their mothers the indicated diet at birth. After weaning, Cldn‐18 KO and heterozygous control mice were kept on their respective diets until 10 weeks of age. The normal‐calcium diet (TD.04200, containing 0.6% calcium carbonate and 0.4% phosphate by weight) and the high‐calcium diet (TD.96348, containing 2% calcium carbonate and 1.25% phosphate by weight) were purchased from Harlan Teklad (Madison, WI).

### Measurement of gastric pH

Gastric pH was assessed in 10 week–old‐mice fed a normal‐calcium diet using a modified method of Waisberg et al. (2005). After the mice were euthanized, the stomach was clamped at the esophageocardial and pylorodoudenal junctions, before being removed, and 0.5 mL sterile water injected into the gastric lumen. The stomach fluid contents were then collected, and gastric pH was measured using a PHR‐146 microcombination pH electrode.

### Total calcium serum measurement

The level of total serum calcium was determined in 10 week‐old‐mice fed either a normal‐calcium or a high‐calcium diet using Stanbio Total Calcium LiquiColor© (Arsenazo III) kit as per manufacturer`s instructions (STANBIO, lab, Boerne, TX).

### Bone densitometry

Total and multiple skeletal site BMD was measured by dual energy X‐ray absorptiometry with a PIXImus instrument (Lunar‐Corp, Madison, WI) at 3, 6, and 10 weeks of age as previously described (Yu et al. 2012). To obtain bone mass parameters of the total body, a region‐of‐interest (ROI) rectangle was moved and resized to cover the whole body, and the animal's head was set as an exclusion zone. Femur, tibia, and lumbar vertebra (LV) 5 BMD were obtained by moving the ROI rectangle to the specific region and resized to cover the assigned area. The precision for the BMD was ±1% for repeat measurements of the same bone several times.

### Micro‐CT analysis

Micro‐CT analysis of LV 5 was carried out using a “vivaCT 40” microCT system (Scanco Medical, Bassers‐drof, Switzerland). Transverse slices were acquired for the entire vertebra body, as previously described (Glatt et al. 2007). Trabecular bone was evaluated in a region 0.3 mm below the cranial and above the caudal growth region. The cortical parameters and cross‐sectional areas were evaluated for 100 slices by delineating a ROI around the entire vertebral body. The thresholds were set at 220 for trabecular bone, and 260 for cortical bone. The length of LV 5 was recorded at the time of scanning.

### Dynamic calcein labeling and histomorphometry

Cldn‐18 KO and heterozygous control mice were injected intraperitoneally with calcein 8 days (20 mg/kg of body weight) and 2 days prior to euthanization in order to label mineralized bone surfaces (BS). LV 5 were fixed in 10% formalin overnight, the bones were washed, dehydrated, and embedded in methyl methacrylate for sectioning. The sections were stained for tartrate‐resistant acid phosphatase (TRAP), and the cortical bone and trabecular bone that adjacent to the growth plate were excluded. The trabecular surface and TRAP‐labeled surface were measured using the OsteoMeasure software (Osteometric, Inc., Decatur, GA) (Xing et al. 2010).

### Cell culture

Primary osteoclast precursors or bone marrow macrophages (BMMs) were isolated from femurs and tibias of Cldn‐18 KO and heterozygous control mice, as described previously (Bradley and Oursler 2008), and maintained in *α*‐minimum essential medium supplemented with 10% fetal bovine serum (FBS), penicillin (100 units/mL), streptomycin (100 μg/mL), and MCSF (25 ng/mL). After 24 h, nonadherent cells (BMMs) were collected and treated with MCSF (50 ng/mL) and RANKL (100 ng/mL) for 6 days; with fresh differentiation media added every 3 days. MCSF and RANKL were obtained from R&D Systems (Minneapolis, MN).

### RNA extraction and gene expression analysis

RNA was extracted using Trizol and chloroform, and isolation was completed using E.Z.N.A.^®^ RNA Isolation Kits (Omega Bio‐Tek, Norcross, GA). The expression was determined by real‐time reverse transcription polymerase chain reaction (RT‐PCR). The housekeeping gene peptidylpropyl isomerase A (PPIA) was used as internal control in the PCR reaction and the ^ΔΔ^CT method was used for relative quantification of gene expression. Values are presented as fold change of control heterozygous mice.

### Statistical analysis

Data were expressed as the mean ± SEM and were analyzed using Student`s t‐test or analysis of variance (ANOVA) (Statistica 6, Tulsa, OK).

## Results

### Cldn‐18 deficiency affects gastric pH

In order to determine the effect of Cldn‐18 on gastric acidity, the stomach pH was measured in both 10‐week‐old Cldn‐18 KO and heterozygous control mice. We found that the lack of Cldn‐18 dramatically increased the gastric pH (pH = 6.1) compared to heterozygous control mice (pH = 2.6) (Table 1).

**Table 1.** The effect of dietary calcium on gastric pH, serum calcium, and lumbar vertebra 5 trabecular and cortical morphology in 10‐week‐old Cldn‐18 KO and heterozygous control mice.

	Normal‐calcium diet		High‐calcium diet		*P* value			
	Control	Cldn‐18 KO	Control	Cldn‐18 KO	Control high vs. control normal	KO high vs. KO normal	KO normal vs. control normal	KO high vs. control high
Gastric pH	2.56 ± 0.08	6.11 ± 0.05	N.D.	N.D.	N.D.	N.D.	^***^	N.D.
Serum Ca (mg/dL)	9.61 ± 0.13	9.26 ± 0.06	10.01 ± 0.07	9.98 ± 0.1	^*^	^***^	^*^	N.S.
Trabecular bone								
BV/TV	0.34 ± 0.01	0.29 ± 0.006	0.33 ± 0.01	0.28 ± 0.007	N.S.	N.S.	^***^	^***^
Connectivity density (mm^−3^)	215.54 ± 12.15	187.38 ± 5.57	223.79 ± 8.51	204.98 ± 8	N.S.	N.S.	^*^	^*^
Tb.N (1/mm)	6.14 ± 0.19	5.46 ± 0.11	6.16 ± 0.18	5.45 ± 0.13	N.S.	N.S.	^**^	^**^
Tb.Th (mm)	0.057 ± 0.0008	0.054 ± 0.0008	0.056 ± 0.001	0.052 ± 0.009	N.S.	N.S.	^**^	^**^
Tb.Sp (mm)	0.171 ± 0.005	0.188 ± 0.004	0.169 ± 0.005	0.187 ± 0.005	N.S.	N.S.	^*^	^*^
Cortical bone								
Total vBMD (mg/cm^3^)	347.91 ± 6.09	323.55 ± 3.69	333.74 ± 6.95	311.605 ± 4.66	N.S.	N.S.	^**^	^*^
Cortical vBMD (mg/cm^3^)	960.91 ± 1.81	941.19 ± 1.66	954.19 ± 3.92	935.27 ± 3.02	N.S.	N.S.	^***^	^**^
Cort. Th (mm)	0.073 ± 0.0001	0.066 ± 0.001	0.071 ± 0.001	0.064 ± 0.001	N.S.	N.S.	^***^	^***^

Data represents the mean ± SEM. *n* = 15–17/group. BV/TV, bone volume/total volume; Tb.N, trabecular number; Tb.Th, trabecular thickness; Tb.Sp, trabecular separation; vBMD, volumetric BMD; Cort. Th, cortical thickness; N.S., not significant; N.D., not determined.

*P < 0.05; **P < 0.01 and ***P < 0.001.

### The effect of dietary calcium on serum calcium levels in 10‐week‐old Cldn‐18 KO and heterozygous control mice

To determine the effect of Cldn‐18 KO on serum calcium levels and verify the effectiveness of giving high calcium in the diet to correct calcium deficit in Cldn‐18 KO mice, we measured total serum calcium levels in Cldn‐18 KO and heterozygous control animals. Expectedly, the high‐calcium diet significantly increased serum calcium levels in both Cldn‐18 KO and heterozygous control mice (Table 1). Interestingly, serum calcium levels were found to be lower in Cldn‐18 mice fed a normal‐calcium diet compared to heterozygous control mice fed the same diet (Table 1). However, serum calcium levels were not different between Cldn‐18 KO and heterozygous control mice on a high‐calcium diet (Table 1).

### The effect of dietary calcium on BMD of the Cldn‐18 KO and heterozygous control mice at different skeletal sites

**Figure 1.**
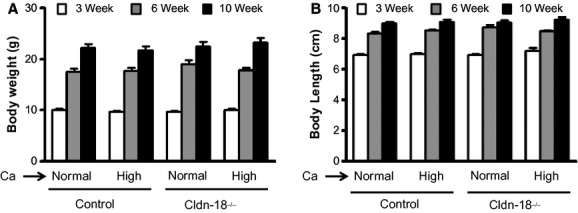
The effect of dietary calcium on body weight and length of Cldn‐18 KO and heterozygous control mice. (A) Body weight of 3‐, 6‐, and 10‐week‐old Cldn‐18 KO and heterozygous control mice (*n* = 13–19/group). (B) Body length of 3‐, 6‐, and 10‐weekold Cldn‐18 KO and heterozygous control mice (*n* = 13–19/group, mixed gender).

**Figure 2.**
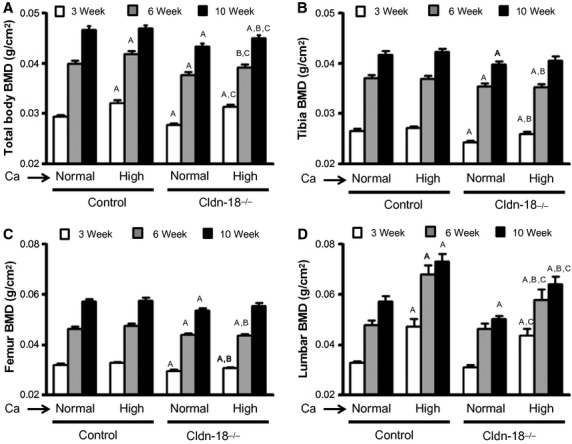
The effect of high‐calcium diet on areal BMD of Cldn‐18 KO and heterozygous control mice at different ages. (A) Total body BMD as determined by dual energy X‐ray absorptiometry at 3, 6, and 10 weeks of age. (B) Tibia BMD. (C) Femur BMD. (D) Lumbar vertebra BMD (*n* = 13–19/group, mixed gender). (A) *P* < 0.05 versus heterozygous control mice (normal‐calcium diet), (B) *P* < 0.05 versus heterozygous control mice (high‐calcium diet), (C) *P* < 0.05 versus KO mice (normal‐calcium diet).

We have previously reported a decrease in bone mass and an increase in BR in Cldn‐18 KO mice (Linares et al. 2012). To evaluate whether these observations are a consequence of decreased calcium absorption due to low‐gastric acidity, Cldn‐18 KO and heterozygous control mice were subjected to either a high‐calcium (2%) or normal‐calcium (0.6%) diet at birth that was continued until 10 weeks of age. First, and as expected, the body weight and animal length increased with age in both genotypes (Fig. 1A and B). However, neither body weight nor body length was significantly different between Cldn‐18 KO and heterozygous control mice fed either a normal‐or high‐calcium diet (Fig. 1A and B). The high‐calcium diet increased total areal BMD in heterozygous control mice compared to a normal‐calcium diet at 3 and 6 weeks of age, by 8% and 5%, respectively (Fig. 2A). A similar trend was observed in the Cldn‐18 KO mice, as a high‐calcium diet increased whole body areal BMD compared to a normal‐calcium diet at all ages (Fig. 2A). Expectedly, the total areal BMD was decreased significantly in Cldn‐18 KO mice fed a normal diet in comparison to heterozygous control mice fed a normal‐calcium diet (Fig. 2A). Moreover, total body areal BMD was significantly lower in the high‐calcium Cldn‐18 KO group compared to the high‐calcium heterozygous control group at 6 and 10 weeks. Therefore, this data suggest that the high‐calcium diet did not rescue the reduced whole body BMD phenotype in the KO mice (Fig. 2A). Phenotypic differences due gender‐genotype‐diet interaction were not observed and, therefore, data from both genders were pooled for analyses. Evaluation of BMD at different skeletal sites revealed that the high‐calcium diet had no significant effect on femur and tibia BMD in both Cldn‐18 KO and heterozygous control mice compared to mice on a normal‐calcium diet, at any age (Fig. 2B and C). However, and as previously documented, Cldn‐18 KO mice exhibited a significant decrease in tibia and femur BMD compared to heterozygous control mice on a normal‐calcium diet (Fig. 2B and C). Moreover, Cldn‐18 KO mice treated with a high‐calcium diet exhibited a significantly lower femur and tibia BMD compared to heterozygous control mice treated with a high‐calcium diet at 3 and 6 weeks of age (Fig. 2B and C). By contrast, the lumbar BMD increased significantly in the high‐calcium heterozygous control group by 44%, 42%, and 27% at 3, 6, and 10 weeks of age, respectively, compared to the normal‐calcium heterozygous control group (Fig. 2D). The same trend was observed in Cldn18 KO mice fed a high‐calcium diet compared to a normal‐calcium diet (Fig. 2D). Even though lumbar BMD was lower in normal calcium fed Cldn‐18 KO mice compared to heterozygous control mice, the difference was not significant until the mice reached 10 weeks of age (Fig. 2D). However, lumbar BMD was significantly lower in the high‐calcium Cldn‐18 KO group compared to the high‐calcium heterozygous control group at 6 and 10 weeks (Fig. 2D). ANOVA indicated that the genotype alone had a significant effect on areal BMD and the age alone had a significant effect on areal BMD (Table 2). Furthermore, areal BMD was significantly affected by diet alone (Table 2). However, there was no significant genotype‐diet‐age interaction for total areal BMD and skeleton‐specific BMD Table 2.

**Table 2.** The effects of genotype, diet, and age on areal BMD using ANOVA analysis.

Grouping variable	Total areal BMD	Tibia BMD	Femur BMD	Lumbar BMD
Genotype	<0.001	<0.001	<0.001	<0.001
Diet	<0.001	<0.001	<0.001	<0.001
Age	<0.001	<0.001	<0.001	<0.001
Genotype + diet	N.S.	N.S.	N.S.	N.S.
Genotype + age	N.S.	N.S.	N.S.	N.S.
Diet + age	<0.05	<0.05	N.S.	N.S.
Genotype + diet + age	N.S.	N.S.	N.S.	N.S.

Data represent *P* values of the interaction of *y*‐axis variables with *x‐*axis parameters calculated using ANOVA. Areal BMD was measured in 3‐, 6‐, and 10‐week‐old Cldn‐18 KO and heterozygous control mice fed either a normal‐ or a high‐calcium diet using Piximus. N.S.: not significant.

### The effect of dietary calcium on LV 5 bone mass parameters of 10‐week‐old Cldn‐18 KO and heterozygous control mice

**Figure 3.**
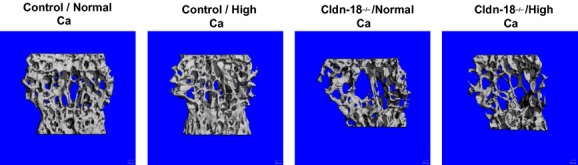
The effect of dietary calcium on lumbar vertebra 5 trabecular morphology. Three‐dimensional *μ*‐CT images of lumbar trabecular bone from 10‐week‐old Cldn‐18 KO and heterozygous control mice.

To further characterize the effect of the calcium diet on the LV, trabecular and cortical bone parameters were evaluated at LV 5 from 10‐week‐old Cldn‐18 KO and heterozygous control mice using *μ*CT. The high‐calcium diet had no significant effect on trabecular bone parameters compared to the normal‐calcium diet in heterozygous control mice (Table 1, Fig. 3). A similar finding was observed in the Cldn‐18 KO mice, as a high‐calcium diet had no effect on trabecular parameters compared to a normal‐calcium diet (Table 1, Fig. 3). However, and as expected, the trabecular bone parameters were found to have significantly deteriorated in Cldn‐18 KO compared to heterozygous control mice fed a normal‐calcium diet (Table 1, Fig. 3). Moreover, Cldn‐18 KO mice treated with a high‐calcium diet exhibited a significantly deteriorated trabecular architecture compared to heterozygous control mice under similar dietary conditions. The same trend was observed for cortical bone parameters (Table 1). Thus, these data provide further evidence that a high‐calcium diet failed to rescue the osteopenia phenotype at LV 5 (Table 1).

### The effect of dietary calcium on LV 5 bone size parameters of 10‐week‐old Cldn‐18 KO and heterozygous control mice

**Figure 4.**
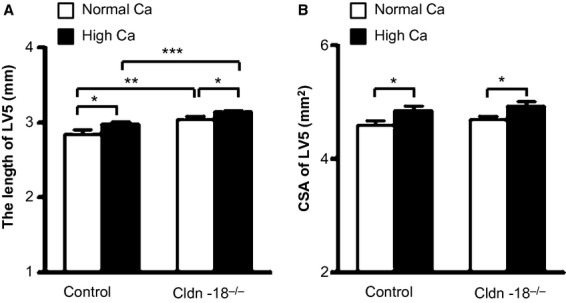
The effect of dietary calcium on bone size parameters of lumbar vertebra 5 (LV5) in 10‐week‐old Cldn‐18 KO and heterozygous control mice. (A) The length of lumbar vertebra 5. (B) The cross‐sectional area (CSA) calculated in the middle region of lumbar vertebra 5 using the *μ*CT (*n* = 12–17/group, mixed gender). *P < 0.05; **P < 0.01 and ***P < 0.001.

As areal BMD is influenced by size parameters, the length of LV 5 was measured to see if the change in lumbar BMD observed with piximus data is due to a size difference. The length of LV 5 was increased by 5% (*P* < 0.05) in the high‐calcium heterozygous control group compared to the normal‐calcium heterozygous control group. A similar increase was also seen in the Cldn‐18 KO mice (Fig. 4A). Furthermore, the cross‐sectional area of LV 5 was also increased significantly in both Cldn‐18 KO and heterozygous control mice fed with high dietary calcium compared to normal dietary calcium (Fig. 4B).

### The effect of dietary calcium on histomorphometric parameters of LV 5 of 10‐week‐old Cldn‐18 KO and heterozygous control mice

**Figure 5.**
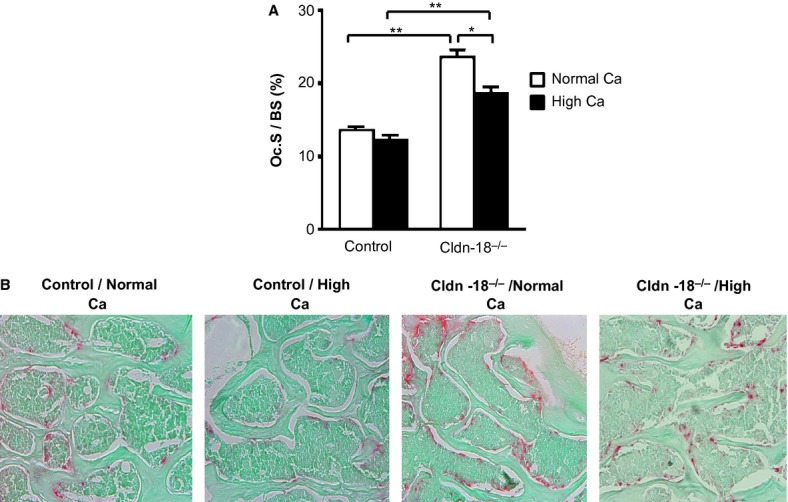
The effect of dietary calcium on lumbar vertebra 5 TRAP‐labeled trabecular surface of Cldn‐18 KO and heterozygous control mice at 10 weeks of age. (A) Osteoclast surface to bone surface (Oc.S/BS) was measured at trabecular bone of lumbar vertebra 5 (*n* = 5–6/group, mixed gender). (B) Representative micrograph of lumbar vertebra 5 sections stained with TRAP (10×). *P < 0.01 and **P < 0.001.

As the lumbar BMD was dramatically increased in both Cldn‐18 KO and heterozygous control mice fed a high‐Ca diet and the lack of Cldn‐18 was previously reported to affect BR, we performed histomorphometric analysis at LV 5 and measured TRAP‐stained surfaces of the trabecular bone. Our analysis showed that the osteoclast surface to BS (Oc.S/BS) was decreased significantly in the high‐calcium Cldn‐18 KO group compared to the normal‐calcium Cldn‐18 KO group (Fig. 5). A similar trend was observed in the heterozygous control mice, as a high‐calcium diet decreased Oc.S/BS compared to a normal‐calcium diet; however, this reduction in Oc.S/BS was not statistically significant, which could be due to either the low baseline resorption of control animals, or the small sample size examined (Fig. 5). Expectedly, Oc.S/BS was increased significantly in Cldn‐18 KO mice fed a normal diet in comparison to heterozygous control mice fed a normal‐calcium diet (Fig. 5). Moreover, Oc.S/BS was significantly higher in the high‐calcium Cldn‐18 KO group compared to the high‐calcium heterozygous control group. Therefore, these findings suggest that the high‐calcium diet did not rescue the increased BR observed in the KO mice.

### The effect of Cldn‐18 deficiency on acid secretion by osteoclasts

**Figure 6.**
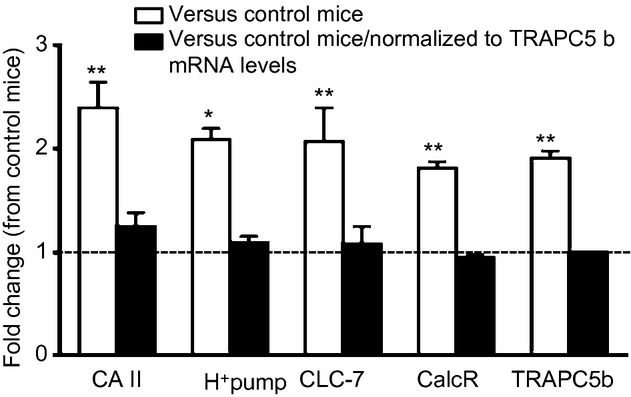
The effect of Cldn‐18 deficiency on the expression of osteoclastic acid secreting machinery. The expression levels of carbonic anhydrase II (CA II), CLC‐7, H^+^ pump, calcitonin receptor (CalcR), and TRAPC5b were determined by real‐time RT‐PCR of mature osteoclasts derived from Cldn‐18 KO and heterozygous control mice. Values are presented as fold change of control heterozygous mice (*n* = 4–5/group). *P < 0.05 and **P < 0.01 versus heterozygous control mice.

To test the possibility that increased BR observed in Cldn‐18 KO mice is due to increased acid secretion from mature osteoclasts, we measured mRNA levels of carbonic anhydrase II, chloride channel‐7 (CLC‐7), and H^+^ pump in primary osteoclasts derived from Cldn‐18 KO and heterozygous control mice. The expression levels of carbonic anhydrase II, CLC‐7, and H^+^ pump was upregulated in Cldn‐18 KO compared to heterozygous control mice (Fig. 6). Because the number of TRAP‐positive multinucleated cells was greater in RANKL/MCSF‐treated BMM cultures derived from Cldn‐18 KO mice (Linares et al. 2012), we normalized mRNA levels in carbonic anhydrase II, CLC‐7, and H^+^ pump to that of TRAPC5b mRNA, a well‐established marker of osteoclast number. Upon normalization to expression levels of TRAPC5b, no significant change was observed in these “normalized” values between Cldn‐18 KO and heterozygous control mice (Fig. 6). Additionally, the expression of calcitonin receptor was also not significantly different between the two genotypes after normalization to TRAPC5b mRNA levels (Fig. 6).

## Discussion

In this study, we found that the loss of Cldn‐18 negatively affected gastric acidity in adult mice. Consistent with this observation, Sanada et al. (2006) have reported that Cldn‐18 was downregulated in gastric cancer and atrophic gastritis. Furthermore, Hayashi et al. (2012) have recently demonstrated that Cldn‐18‐deficient mice developed atrophic gastritis and the gastric pH was significantly higher in Cldn‐18 KO mice compared to WT mice at day 14 postnatally. Additionally, these authors found that the H^+^ leakage into the submucosal layer of gastric tissues was higher in Cldn‐18 KO mice compared to WT mice, and was associated with the upregulation of proinflammatory cytokines which in turn induced gastritis (Hayashi et al. 2012; Tamura et al. 2012). Furthermore, the expression of H^+^‐K^+^ ATPase was found to be downregulated in Cldn‐18 KO mice, which is caused by a decrease in the total number of well‐differentiated parietal and chief cells (Hayashi et al. 2012). Because Cldn‐18 is a novel negative regulator of BR, we tested the possibility that Cldn‐18 deficiency affects acid secretion machinery in osteoclasts. We did not find significant differences in the expression levels of any of the three markers of acid secretion between Cldn‐18 KO and heterozygous control mice after adjustment for differences in osteoclast number using TRAPC5b mRNA levels. Based on these and published data, it appears that the increased gastric pH (due to less acid secretion) observed in Cldn‐18 KO mice is due to a defect in the H^+^‐resistant paracellular barrier between gastric epithelial cells and a decrease in the total number of parietal cell induced by inflammation, while the increased BR in Cldn‐18 KO mice is due to MCSF/RANKL signaling‐induced increase in osteoclast number and not due to increased acid secretion per se per osteoclast. Further studies are, however, needed to verify these conclusions.

It is noteworthy that the role of gastric acidity in calcium absorption/metabolism has received more attention recently (Wright et al. 2008; Boyce 2009). Calcium solubilization is thought to be prerequisite for calcium absorption in the small intestine, with the stomach's acidic environment inducing the dissolution of calcium salts and the release of ionized calcium (Bo‐Linn et al. 1984; Sipponen and Harkonen 2010). To this end, several clinical studies reported a positive association between the long‐term use of acid suppressing agents and bone fractures (Vestergaard et al. 2006; Yang et al. 2006; Ito and Jensen 2010). Moreover, short and long‐term use of acid suppressing agents decreased BMD (Adachi et al. 1998; Kinjo et al. 2008). Although there is limited experimental evidence regarding the mechanism of acid suppressing agent‐induced bone loss, it seems to be more related to the inhibitory effect of acid on calcium absorption (Wright et al. 2008). Recently, Schinke et al. (2009) have reported that mice deficient in cholecystokinin B‐gastrin receptor that affects acid secretion by parietal cells, exhibited an osteoporotic phenotype, mediated by an alteration in calcium homeostasis. These authors also found that a high‐calcium diet fully rescued hypochlorhydria‐induced bone loss in these mice. Similarly, a high‐calcium diet is routinely used in the literature to correct calcium deficit induced by gene mutations (Amling et al. 1999; Dardenne et al. 2003; Schinke et al. 2009). The efficacy of the dietary approach we employed in this study was in fact reported and validated in several animal model studies in which calcium absorption and homeostasis were perturbed. For example, the bone phenotype of vitamin D receptor‐deficient mice was completely rescued by feeding them a high‐calcium diet (Amling et al. 1999; Dardenne et al. 2003). Additionally, several studies have demonstrated that feeding pregnant mothers with a high‐calcium diet significantly increases calcium content in the milk, thereby providing an effective means to correct calcium deficiency in the pups (Cao et al. 2009; Shu et al. 2011). Furthermore, our data that areal BMD of vertebra is increased in high‐calcium diet groups of both genotypes and that serum calcium levels were significantly higher in high‐calcium diet groups compared to normal‐calcium diet groups are in support of the notion that the approach that we used was effective in correcting calcium deficit in the Cldn‐18 KO mice.

In agreement with our previous studies in which the serum PTH levels were found to be elevated in Cldn‐18 KO mice fed a normal‐calcium diet compared to control mice, we found the serum calcium levels were lower in Cldn‐18 KO mice compared to heterozygous control mice fed a normal‐calcium diet. In addition, the serum calcium levels were significantly higher in Cldn‐18 KO mice fed a high‐calcium diet compared to a normal‐calcium diet. Moreover, BR (Oc.S/BS) was lower in high‐calcium diet fed groups compared to normal‐calcium diet fed groups. Together these results suggest that Cldn‐18 KO mice may be calcium deficient and are in support of the possibility that the high systemic calcium achieved by high‐calcium diet was effective in correcting the calcium deficit in Cldn‐18 KO mice. Importantly, the finding that the high dietary calcium did not rescue the osteopenia phenotype in Cldn‐18 KO mice supports the idea that low gastric acidity may not be responsible for the Cldn‐18 deficiency‐mediated bone loss. In this regard, we have recently obtained evidence suggesting that the low BMD observed in Cldn‐18 KO mice may derive from the lack of Cldn‐18 in bone cells. Specifically, we have shown that Cldn‐18 is expressed in osteoclasts and that lack of Cldn‐18 increased osteoclast differentiation and BR (Linares et al. 2012). Taken together, future studies will elucidate the direct role of Cldn‐18 on bone cells using mice with a conditional disruption of Cldn‐18 in osteoclasts.

We also found that various skeletal sites respond differently to increased dietary calcium. Thus, a high‐calcium diet increased lumbar BMD in both Cldn‐18 KO and heterozygous control mice compared to their corresponding genotypes on a normal‐calcium diet, whereas tibia and femur BMD were unaffected by dietary calcium in both genotypes. Consistent with our finding, Datta et al. (2010), showed an increase in LV 5 BMD in both WT and phosphorylation deficient PTH1R knock‐in mice fed a high‐calcium diet starting at 4 week of age that was stopped at 8 week of age compared to the genotype‐matched mice fed a normal‐calcium diet. On the other hand, these authors observed that femur BMD was unaffected by a high‐calcium diet in both of these genotypes (Datta et al. 2010). To our knowledge, our study is the first to document a positive association between spine BMD and a high‐calcium diet that is started at birth, in mice. In contrast to our results, it has been previously reported that tibia BMD increased in 3‐week‐old WT and PTH‐deficient mice fed by dams who received a high‐calcium diet compared to those on a normal‐calcium diet (Cao et al. 2009; Shu et al. 2011). Skeletal site selectivity in response to calcium supplementation is well‐documented in human studies during childhood and adolescence, but the mechanism of this site‐specific effect remains to be elucidated (Bonjour et al. 1997; Chevalley et al. 2005).

Although lumbar BMD increased significantly in response to a high‐calcium diet in both Cldn‐18 KO and heterozygous control mice, we did not find a significant change in either the trabecular or cortical bone parameters at LV 5, as measured by *μ*CT. One explanation is the size difference in the LV 5 between the high‐calcium diet group and the normal‐calcium diet. We also found that the loss of Cldn‐18 in mice fed a normal‐calcium diet decreased the total body BMD, trabecular, and cortical bone parameters, which was consistent with our previous report on the Cldn‐18‐deficient mice bone phenotype (Linares et al. 2012). However, increased dietary calcium intake in Cldn‐18‐deficient mice did not rescue this phenotype at different skeletal sites. Furthermore, the lumbar BR (Oc.S/BS) was still significantly higher in Cldn‐18 KO mice fed a high‐calcium diet compared to heterozygous control mice fed a high‐calcium diet. Collectively, correction of serum calcium deficit did not correct decreased BMD and increased BR observed in Cldn‐18 KO mice, thus ruling out the possibility that gastric abnormalities contributed to the osteopenia phenotype in these mice. While these data suggest a direct role of Cldn‐18 in osteoclasts, more direct evidence from the use of osteoclast‐specific Cldn‐18 KO mice are warranted to convincingly demonstrate a noncanonical role of Cldn‐18 in osteoclasts.

The limitations of this study are as follows. First, we did not measure the impact of changes in gastric pH in Cldn‐18 KO mice on calcium absorption by directly measuring dietary calcium uptake using calcium isotopic tracers. Second, we used heterozygous mice lacking one functional allele of Cldn‐18 based on the earlier finding that skeletal phenotype was not different between WT and heterozygous mice (Krause et al. 2008) and based on the homozygous KO X heterozygous breeding strategy, which yielded 50% KO and 50% heterozygous mice that were used as control. Third, we have not provided direct evidence using mice with conditional KO of Cldn‐18 in osteoclasts to rule out any role for Cldn‐18 expressed in the stomach for the osteopenia phenotype in Cldn‐18 KO mice. Fourth, we did not measure serum PTH and calcitonin levels to check whether the enhanced lumbar BMD and decreased BR observed in both genotypes in response to high dietary calcium were due to either suppression of PTH and/or due to secretion of calcitonin. In this regard, the calcitonin receptor expression was not different after adjustment for differences in TRAP‐positive osteoclasts between the two genotypes.

In conclusion, we demonstrated that Cldn‐18 deficiency negatively affects gastric acidity. In addition, we found that a high‐calcium diet increased lumbar BMD and decreased BR in both Cldn‐18 KO and heterozygous control mice, whereas correcting the deficit in serum calcium in Cldn‐18 KO by feeding a high‐calcium diet did not correct the osteopenia phenotype and the increase in BR. Therefore, these data suggest that the osteopenia phenotype observed in Cldn‐18 KO mice is related to the changes in BR rather than a deficit in calcium absorption.

## Acknowledgments

We thank Robert Brommage and David R. Powell at Lexicon Pharmaceuticals Inc. for providing Cldn‐18 KO mice for our studies. We thank Nancy Lowen for technical assistance.

## Conflict of Interest

None declared.
